# Supplementary material for: FGF-2 Differentially Regulates Lens Epithelial Cell Behaviour during TGF-β-Induced EMT
Source: Cells. 2023 Mar 7;12(6):827. doi: 10.3390/cells12060827 (PMC10046997; doi:10.3390/cells12060827)
Supplement: Supplementary file 1 [file cells-12-00827-s001.zip › cells-2250599-supplementary.pdf]

Supplementary Figures

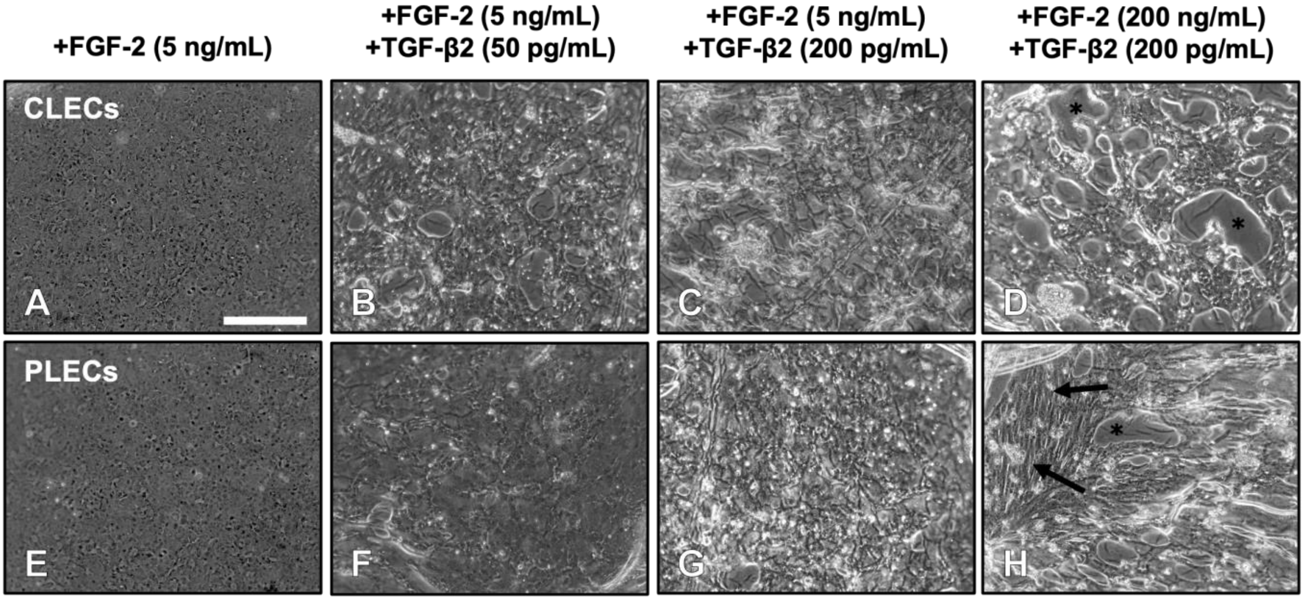

Supplementary Figure S1. FGF dose-dependent modulation of TGF-β2-induced EMT. Representative images of LEC explants treated with a low dose of FGF-2 (5 ng/mL) at 3 days of culture (A, E). Explants treated with low dose FGF-2 and low dose TGF-β2 (50 pg/mL) underwent an EMT response in central (CLECs, B) and peripheral regions (PLECs, F). A low dose of FGF-2 and high dose of TGF- β2 (200 pg/mL) led to an EMT response in both CLECs (C) and PLECs (G). A high dose of FGF-2 (200 ng/mL) and TGF-β2 (200 pg/mL) also led to an EMT response in both CLECs and PLECs (D, H), with prominent myofibroblastic cells (H, arrows) and increased modulation/wrinkling of the exposed underlying lens capsule (asterisks). Scale bar: 200 μm.

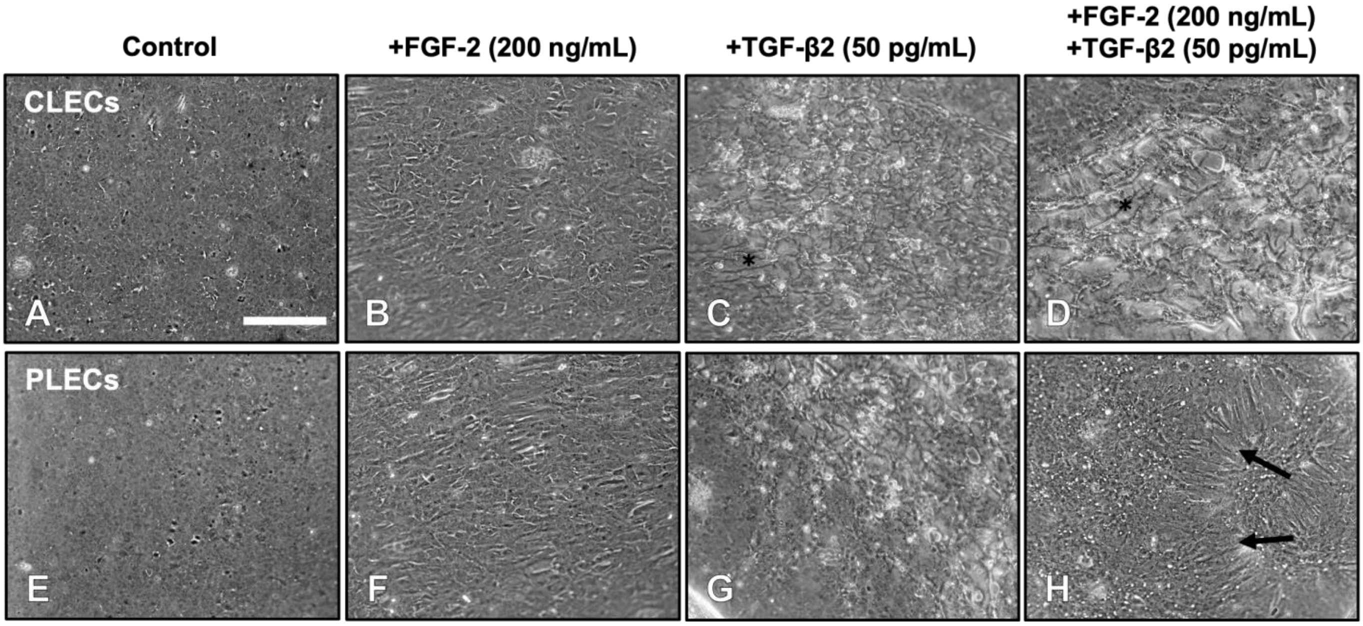

Supplementary Figure S2. FGF-2 modulates LEC behaviour during TGF-β2-induced EMT. Representative image of control explants with a tightly packed cobblestone-like epithelium over 3 days (A, E). Treatment with FGF-2 promotes elongation and lens fibre differentiation (B, F). TGF-β2 induces EMT throughout the explant (C, G), with cells acquiring contractile properties and modulating the underlying lens capsule (asterisks). FGF-2/TGF-β2 cotreated explants exhibited an EMT response in CLECs (D), but a fibre differentiation response in PLECs (H, arrows). Scale bar: 200 μm.

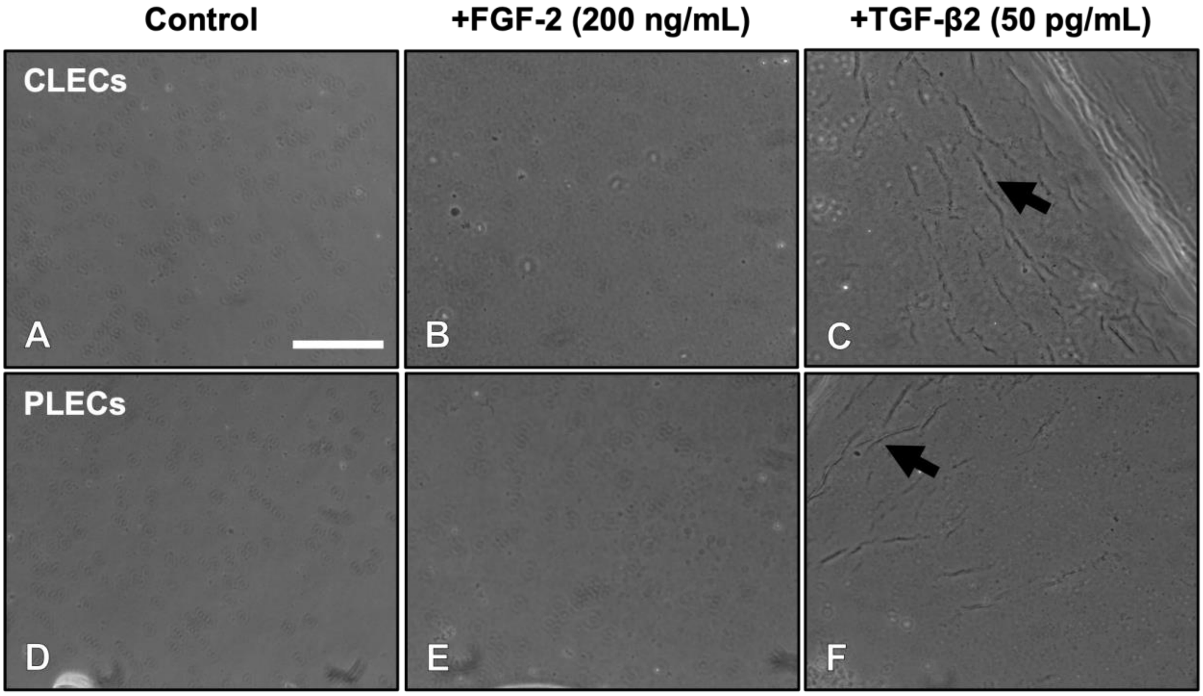

Supplementary Figure S3. Lens capsule modulation. 72 hours post-treatment, explants were rinsed consecutively in filtered Milli-Q H_2O to remove all lens epithelial cells and view the underlying lens capsule. No capsular wrinkling was evident in control (A, D) and/or FGF-2 treated (B, E) explants. Capsular modulation was evident in TGF-β2 treated explants in both central (C) and peripheral (F) cell regions with increased wrinkling (arrowheads). Scale bar: 200 μm.

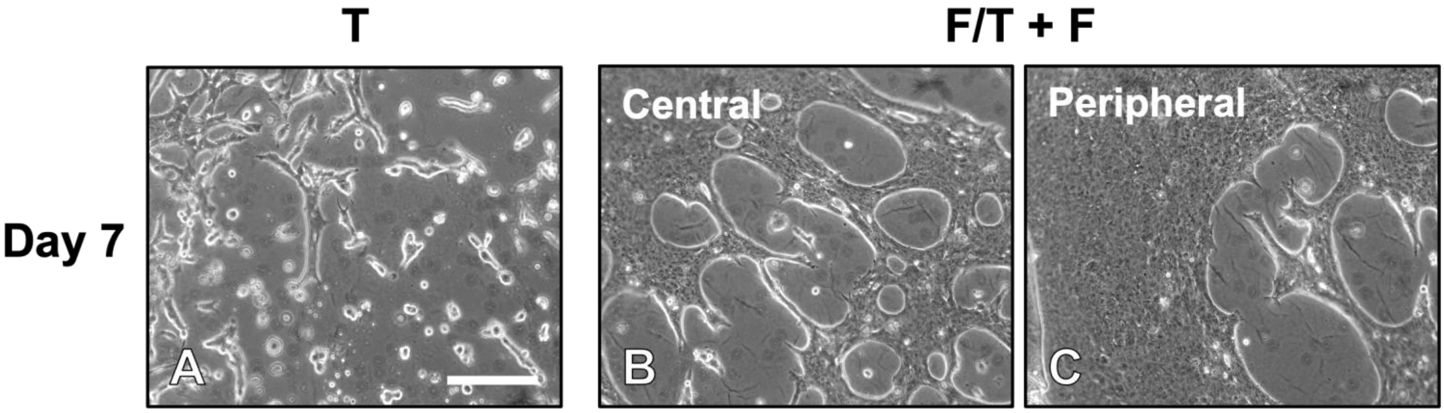

Supplementary Figure S4. Re-supplementation of FGF-2 promotes cell survival**.** A) Representative image of a wholemount explant treated with a high dose of TGF-β2 (200 pg/mL, T), undergoing an EMT response and cell death by day 7 of culture. Re-supplementation with FGF-2 in explants cotreated with a high dose of FGF-2 (200 ng/mL, F) and low dose of TGF-β2 (50 pg/mL, t) after 3 days of culture, promotes cell survival in both central (B) and peripheral (C) lens cells after 7 days. Scale bar: 200 μm.

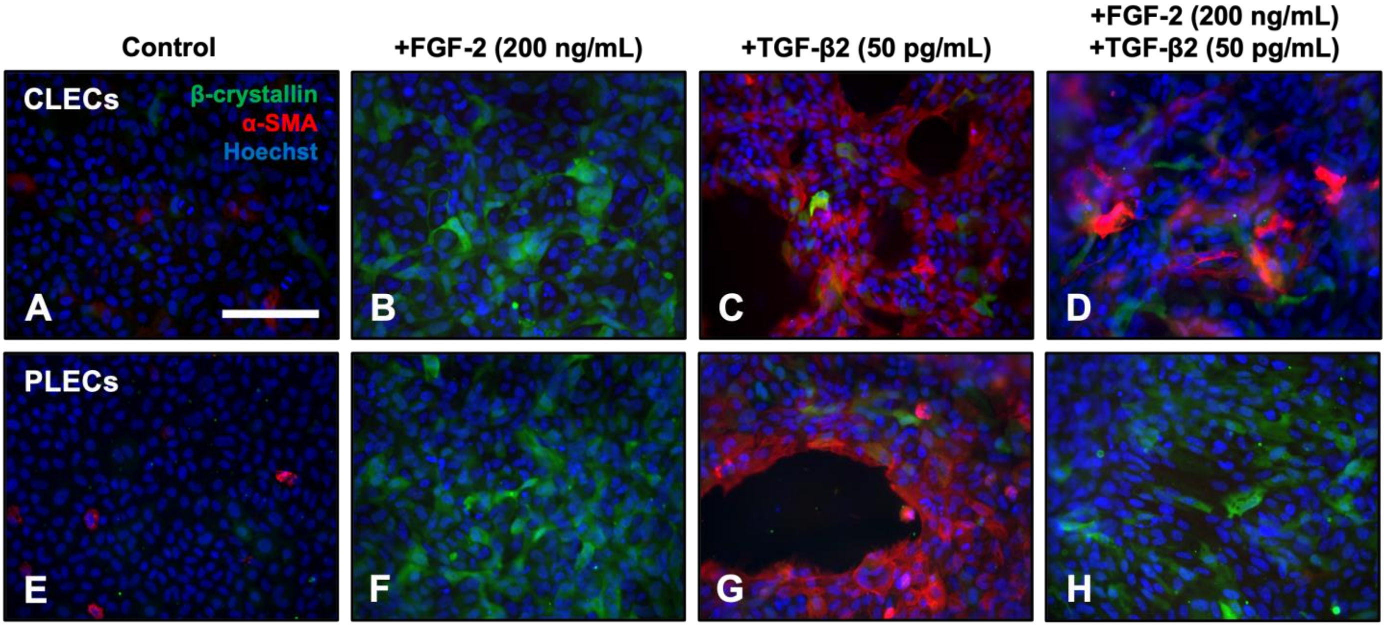

Supplementary Figure S5. FGF-2 modulates EMT and fibre differentiation markers in TGF-β2-treated LECs. Representative immunolabelling for β-crystallin (green), and α-SMA (red), counter-stained with Hoechst nuclear dye (blue), in CLECs (A-D) and PLECs (E-H) following 5 days of culture with no growth factors (Control, A, E), FGF-2 (200 ng/mL; B, F), TGF-β2 (50 pg/mL; C, G), or cotreated with FGF-2/TGF-β2 (D, H). Scale bar: 100 μm.
